# Voxelotor versus other therapeutic options for sickle cell disease: Are we still lagging behind in treating the disease?

**DOI:** 10.1002/hsr2.713

**Published:** 2022-06-21

**Authors:** Syeda Tayyaba Rehan, Hassan ul Hussain, Farheen Malik, Rana Muhammad Usama, Muhammad Junaid Tahir, Muhammad Sohaib Asghar

**Affiliations:** ^1^ Department of Medicine Dow University of Health Sciences Karachi Pakistan; ^2^ Lahore General Hospital Lahore Pakistan; ^3^ Department of Internal Medicine Dow University of Health Sciences—Ojha Campus Karachi Pakistan

**Keywords:** blood, drugs, hemoglobinopathies, hydroxyurea, pharmacology, safety

## Abstract

**Background:**

Sickle cell disease (SCD) is one of the most prevalent hemoglobinopathies that affects around 275,000 neonates annually. Until 2017, hydroxyurea was the only available drug for SCD treatment. Later on, L‐glutamine and crizanlizumab have shown promising results in SCD therapy.

**Objectives:**

There were limited pharmacological options for the disease when in November, 2019, voxelotor was approved for the treatment of SCD patients after showing promising results in the clinical HOPE trial. Despite its favorable results, some life‐threatening side effects were also observed. Uncertainty regarding the use of available pharmaceutical therapies for SCD is the major hurdle for the survival of patients.

**Discussion & Conclusion:**

An immediate attention needs to be drawn towards the drawbacks of limited pharmacological options for SCD. Article calls out to conduct more extensive trials in this advanced era of medicine where ambiguity regarding the use of SCD drugs still prevails.

Sickle cell disease (SCD) is one of the most common autosomal recessive hemoglobinopathies, affecting around 275,000 neonates worldwide per annum.^1^ Study authors predict that by the year 2050, the prevalence of the disease will increase by 25% and more than 400,000 babies will be born annually with this disorder.^2^

In developed countries, the life expectancy of SCD patients is around 45−50 years. It is even less in low‐ and middle‐income countries, and most patients do not survive until adulthood.^3^ According to an estimate, 50%–80% of the infants born in African countries with SCD die before the age of 5 years.^1^

SCD is a hematological disorder that occurs due to a missense point mutation of valine for glutamine at chromosome 11, supervened by the formation of defected beta chains.^2^, ^3^ This impairment in hemoglobin (Hb) composition ultimately leads to its polymerization and sickling of red blood cells when exposed to risk factors like hypoxemia, acidosis, or loss of body volume.^3^ The significant sequelae of red blood cells sickling are vaso‐occlusive crisis which presents with severe pain and hemolytic anemia.^3^

The onset of complications in SCD patients starts surfacing in childhood, including leg ulcers, anemia, kidney damage, gallstones, stroke, increased risk of infections, and sepsis due to auto splenectomy.^2^ Every day more than 500 children with SCD die because of poor access to appropriate treatment options.^3^ Survival of children having SCD can be increased with proper neonatal screening, early diagnosis of disease, prophylactic penicillin implementation, and immunizations against encapsulated bacteria.^1^

Previously, the burden of SCD was concentrated mainly in the Mediterranean countries and sub‐Saharan deserts. Due to the noticeable advancement in migration, SCD is rapidly propagating to developed countries like Western Europe, South, and East America that previously had low incidences of the disease.^2^

Despite the disease's high prevalence and poor survival rates, hydroxyurea was the only accepted pharmacological option for the treatment of SCD, until 2017.^4^ Hydroxyurea is a ribonucleotide reductase inhibitor that works by increasing the fetal hemoglobin concentration in blood.^5^ It reduces the frequency of pain crises via significant suppression in ischemic and infarctive events of disease. Hydroxyurea essentially replaces the high‐risk blood transfusion therapies routinely performed in SCD patients and is reported safe for long‐term use.^4^ However, due to the myelosuppressive activity of this drug, close monitoring is highly required during treatment. Marrow suppression can further lead to aplastic crisis and weakened immune response if superimposed by infections.^6^

Some highly relevant concerns are being raised regarding the long‐term effects of hydroxyurea, its potential effects on fertility, risks on pregnancy such as teratogenicity, and concern for increased risk of malignancy in recipients.^4^ Based on animal experimental studies of the drug, it is suggestive to discontinue the drug during pregnancy but the effects of hydroxyurea on developing fetus and pregnancy are still not determined and need more understanding.^5^

In 2017, l‐glutamine, an amino acid was approved for the treatment of SCD patients who are older than age 5. l‐glutamine reduces the episodes of vaso‐occlusion, chronic pain, and ultimately decreases the hospitalization rate.^5^ It is a well‐tolerated drug with very mild adverse effects. A clinical trial by Niihara et al.^7^ reported 5 out of 151 patients in the l‐glutamine group withdrew from the therapy because of adverse events including hypersplenism, abdominal pain, dyspepsia, a burning sensation in the feet, and hot flashes. There is no study on the safety of l‐glutamine use in pregnant women and the drug has not yet shown benefits in managing other SCD‐related complications due to its use for a relatively short period.^5^

Crizanlizumab, after receiving Food and Drug Administration (FDA) breakthrough for the prevention of vaso‐occlusive crises in January 2019, was granted FDA approval on November 15, 2019. It is a humanized anti‐P‐selectin antibody that blocks the substrate binding to P‐selectin glycoprotein ligand 1. Inhibition of P‐selectin minimizes the adhesion of sickle erythrocytes and leukocytes to endothelial cells and improves microvascular blood flow velocities.^5^ As a therapeutic monoclonal antibody, crizanlizumab exposes the recipients to risks of infections, which needs to be considered in post‐marketing pharmacovigilance.^8^ Some common adverse outcomes of the drug that have been noticed in more than 10% of the recipients include headache, back pain, arthralgia, diarrhea, vomiting, and pyrexia.^9^ Crizanlizumab was also observed to cause single‐occurrence life‐threatening adverse events such as anemia and intracranial hemorrhage when administered in low doses.^9^

Crizanlizumab as a treatment option for SCD failed to suppress the hemolytic events of the disease. A randomized double‐blind Phase 2 trial by Ataga et al. stated that no significant differences were observed between the active treatment and placebo group in terms of hemolytic crisis.^9^ Further that, no significant improvements in the hemoglobin levels of the recipients were recorded by l‐glutamine and crizanlizumab therapy in different trials.

Because only three medications were FDA‐approved to treat SCD before 2021, the pharmaceutical options were limited. On November 25, 2019, voxelotor, after showing promising results in the clinical HOPE trial, was granted approval for the treatment of SCD patients above or at age 12.^10^

Voxelotor (also known as Oxbryta®) is an orally administered drug that works by inhibiting hemoglobin S (HbS) polymerization. Voxelotor is a first in a class of Hb oxygen affinity modifiers which reversibly and covalently binds with the N‐terminal valine of alpha chains of Hb.^11^ An upsurge in the Hb concentration of blood by 1 g/dl and truncation of hemolytic events were observed within 2 weeks of its first dose.^10^, ^12^ It reduces the sickling of RBC by bringing down the hypoxemic events, which further recedes the hemolysis and vaso‐occlusive infarction in SCD patients (Figure 1).^12^ As monitored in a clinical trial, voxelotor proved to be a well‐tolerated drug up to a dose of 2800 mg, while the most suitable dose suggested for the SCD patients without any comorbidities was 1500 mg once daily.^11^

**Figure 1:**
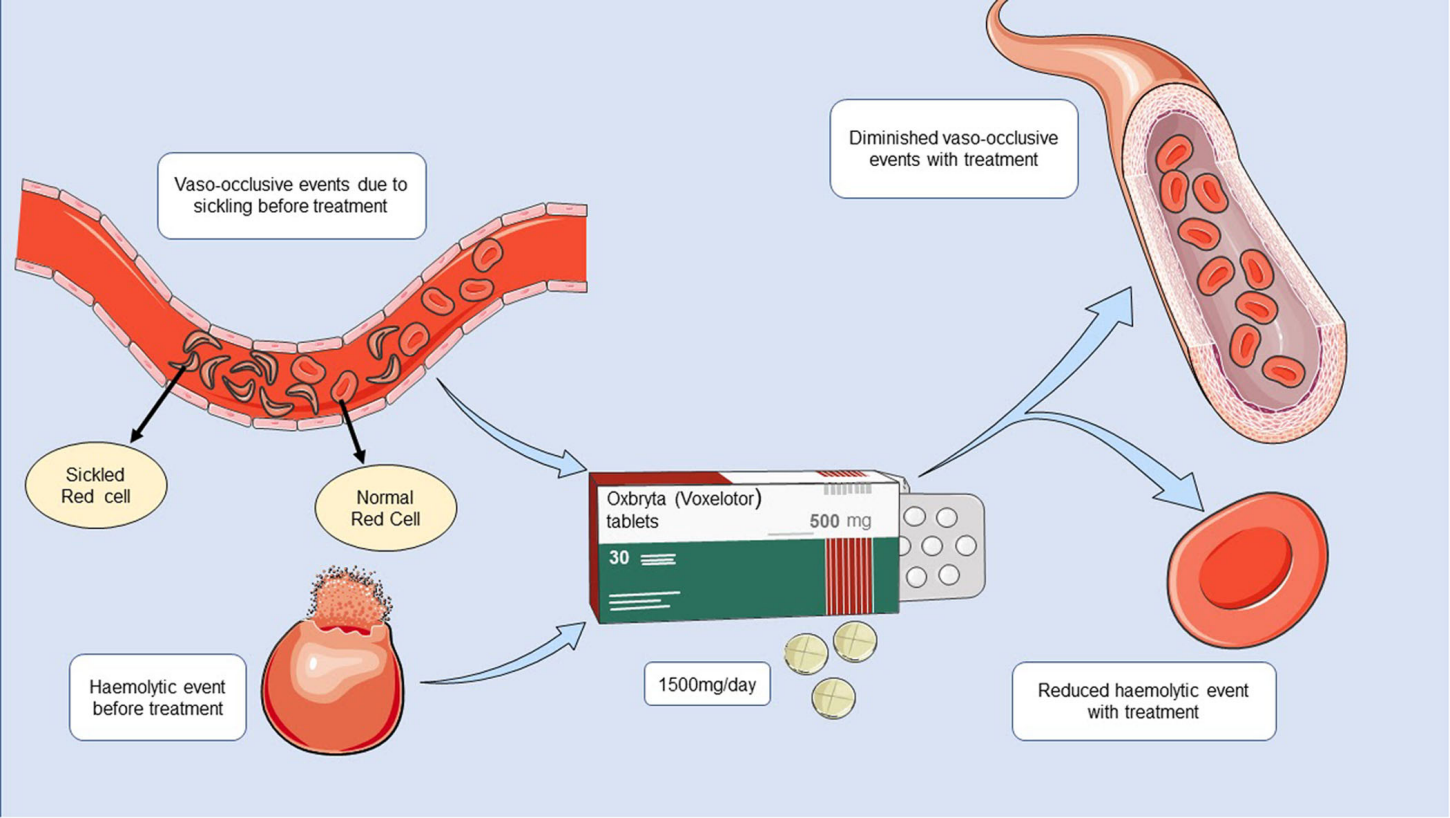
Hemolytic and sickling events of SCD before and after voxelotor administration. SCD, sickle cell disease.

A study of the drug's pharmacokinetics by Hutchaleelaha et al. indicated that the drug gets distributed relatively fast to RBCs once absorbed in plasma. It has a half‐life of 65−85 h in healthy volunteers and around 50 h in SCD patients.^11^ Analysis of the drug's metabolism reveals that voxelotor is majorly metabolized by the liver, so patients with hepatic dysfunction are advised lower doses of this drug (1000 mg).^13^ With minimum renal excretion, the drug can be safely administered in patients with renal impairments.^13^, ^14^

This novel drug was observed to significantly reduce anemia and its hemolytic markers, such as hyperbilirubinemia and lactate dehydrogenase, without affecting the blood viscosity.^12^ Unlike the previous theoretical Hb modifiers, voxelotor does not cause any significant increase in erythropoietin or reticulocytosis.^11^ In a clinical analysis of voxelotor on SCD patients, side effects predominantly noticed during the treatment period were diarrhea, headache, abdominal pain, nausea, vomiting, and the rash of maculopapular variety.^11^ Headache and diarrhea were some of the most prominent side effects.^11^ The majority of volunteers in the trial were reported with mild or moderate symptoms, while only very few (4 out of 81 patients) were recorded to have fatal adverse events during the trial (pulmonary sepsis, sickle cell anemia with crisis, and acute sickle hepatic crisis).^11^, ^12^, ^14^

According to the FDA, voxelotor is contraindicated in patients with a history of serious drug hypersensitivity or patients taking fluconazole, ketoconazole, and rifampin.^15^ Coadministration of voxelotor with CYP34A inhibitors like fluconazole should be avoided as these drugs may lead to its toxicity.^15^ In addition, voxelotor administration was discerned to interfere with the laboratory measurement of Hb subtypes (HbA, HbS, and HbF) by high‐performance liquid chromatography.^15^ Further on, it was clinically considered that voxelotor should only be used during pregnancy if the benefits outweigh the potential risk, as the adverse outcomes of the drug are strongly expected to threaten both the fetus and mother. Breastfeeding is also recommended to be discontinued during treatment and at least 2 weeks after the last dose, considering the possibility of serious adverse outcomes in breastfed children.^15^

Uncertainty regarding the use of available pharmacological drugs for SCD might be one of the reasons for its poor survival rates and under‐5 age deaths. Newly FDA‐approved voxelotor offers advanced therapeutic options for patients with SCD in this exciting era of sickle cell pharmacotherapeutics. Voxelotor promising trials make this drug very important for the treatment of SCD. It appears to be safe both as a single drug or in combination with hydroxyurea for the treatment of SCD. A thorough review of literature gives strong evidence to replace high‐risk therapy like blood transfusion with this novel drug. However, side effects like pulmonary sepsis, hypersensitivity reaction, and anemia with crisis should also be taken into consideration. Therefore, we suggest using appropriate caution before starting this treatment, ensuring a multidisciplinary analysis of benefits and harms.

Given the limited data available, more extensive controlled studies are desperately needed to explore this drug's efficacy and safety especially in pediatric patients as well as in pregnancy with ongoing studies. Moreover, the logistics of voxelotor preparation and administration need to be optimized to improve patient access. We call out for more advanced trials to be conducted for in‐depth evaluation of limited therapeutic options that are available for SCD. Also, continuous reviews and further investigations are necessary to disseminate the latest information on the drug.

## AUTHOR CONTRIBUTIONS

**Syeda Tayyaba Rehan**: Conceptualization; data curation; investigation; visualization; writing—original draft. **Hassan ul Hussain**: Conceptualization; investigation; resources; visualization; writing—original draft. **Farheen Malik**: Data curation; methodology; software; writing—original draft. **Rana Muhammad Usama**: Data curation; investigation; visualization; writing—original draft. **Muhammad Junaid Tahir**: Methodology; project administration; resources; validation; writing—review and editing. **Muhammad Sohaib Asghar**: Formal analysis; software; supervision; validation; writing—review and editing. All authors have read and approved the final version of the manuscript. The corresponding author had full access to all of the data in this study and takes complete responsibility for the integrity of the data and the accuracy of the data analysis.

## CONFLICT OF INTEREST

The authors declare no conflict of interest.

## TRANSPARENCY STATEMENT

Muhammad Sohaib Asghar affirms that this manuscript is an honest, accurate, and transparent account of the study being reported; that no important aspects of the study have been omitted; and that any discrepancies from the study as planned (and, if relevant, registered) have been explained.
